# Pancreatoduodenectomy co-morbid with celiac axis compression syndrome: a report of three cases

**DOI:** 10.1186/s40792-020-00878-x

**Published:** 2020-05-24

**Authors:** Katsuki Miyazaki, Yuji Morine, Yu Saito, Shinichiro Yamada, Kazunori Tokuda, Tetsuya Ikemoto, Satoru Imura, Mitsuo Shimada

**Affiliations:** The Department of Digestive and Transplant Surgery, Tokushima University, 3-18-15 Kuramoto-cho, Tokushima City, Tokushima, 770-8503 Japan

**Keywords:** Pancreatoduodenectomy (PD), Celiac axis compression syndrome (CACS), Median arcuate ligament syndrome (MALS), Gastroduodenal artery (GDA)

## Abstract

**Background:**

Celiac axis compression syndrome (CACS) is a relatively rare disease. Because of the nature of the blood flow in the celiac region when a pancreatoduodenectomy (PD) is performed for CACS, the celiac region can become ischemic. The aim of this study is to report on the importance of pre-operative diagnosis of CACS in terms of the outcomes for patients post-operatively. In this study, three 3 cases of PD co-morbid with CACS are reported: one intra-operative diagnosis case and two pre-operative diagnosis cases.

**Case presentation:**

The one case, not diagnosed with CACS prior to the operation, had a hard post-operative course because of complication caused by ischemia of the celiac region compared with the two cases diagnosed prior to the operation, who had a good post-operative course because of pre-operative or intra-operative intervention.

**Conclusions:**

Post-operative complications due to CACS are preventable by pre-operative diagnosis and appropriate interventions.

## Background

Celiac axis compression syndrome (CACS) is a relatively rare disease caused by stenosis of the celiac trunk. Various symptoms are associated with CACS such as abdominal pain occurring after meals, weight loss, nausea, vomiting, and diarrhea. The causes of CACS are compression by the median arcuate ligament (55%) which is the most common cause and known as median arcuate ligament syndrome (MALS), sclerosis (15%), and others (35%) [1]. In the case of CACS, the blood flow in the celiac region, including the hepatic artery, is preserved by the flow of the gastroduodenal artery (GDA) from the superior mesenteric artery. So when a pancreatoduodenectomy (PD) with the dissection of GDA is performed, the celiac region can become ischemic. Ischemia of the celiac region causes high mortality complications such as liver failure, liver abscess, and anastomotic dehiscence with subsequent bile leakage [1–4]. It is reported that about 2 to 7.6% of cases of PD are co-morbid with CACS [1–3, 5]. Recently, Japanese Society of Hepato-Biliary-Pancreatic Surgery obligates board-certified expert surgeons to perform intraoperative GDA clump test in PD. In this study, three cases of PD co-morbid with CACS are reported, and the literature, including the most recent available, is introduced.

## Case presentation 1

**Fig. 1:**
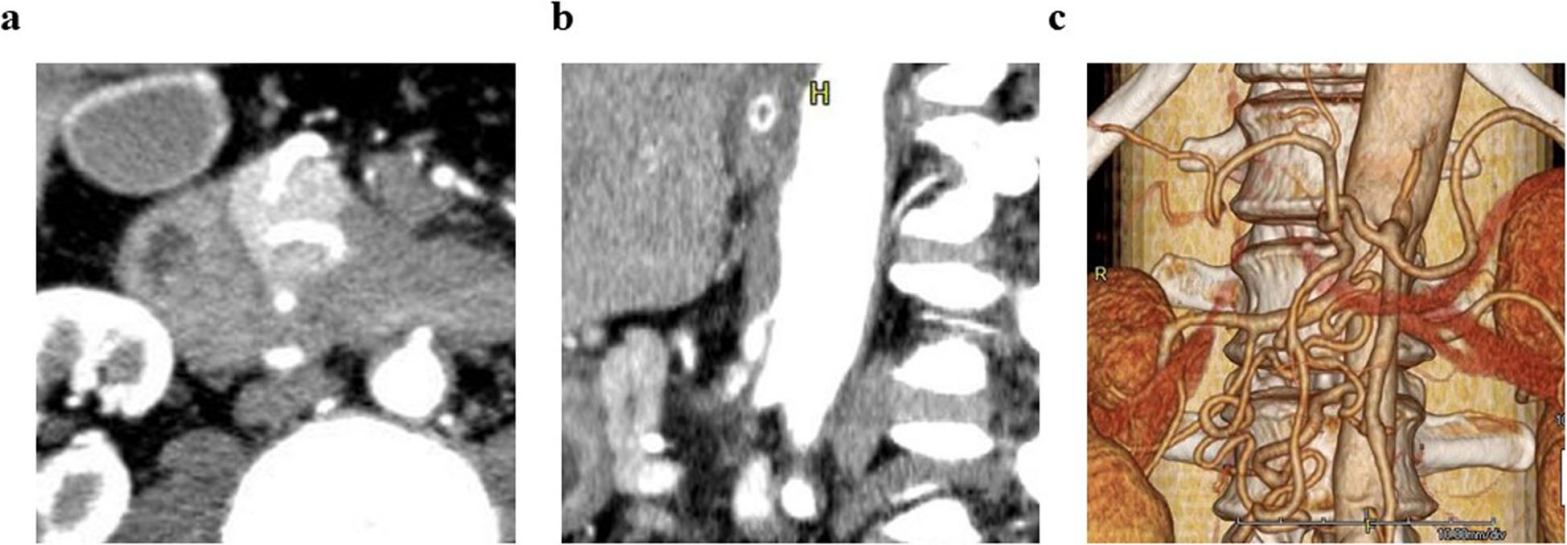
Preoperative enhanced CT (case 1). **a** Low-density area surrounding normal tissue of the pancreas and duodenum. **b** CACS due to MALS. Hooked appearance and minimal post-stenotic dilatation. **c** 3D reconstruction image of blood vessels. Inferior pancreaticoduodenal artery was dilated and tortuous

**Fig. 2:**
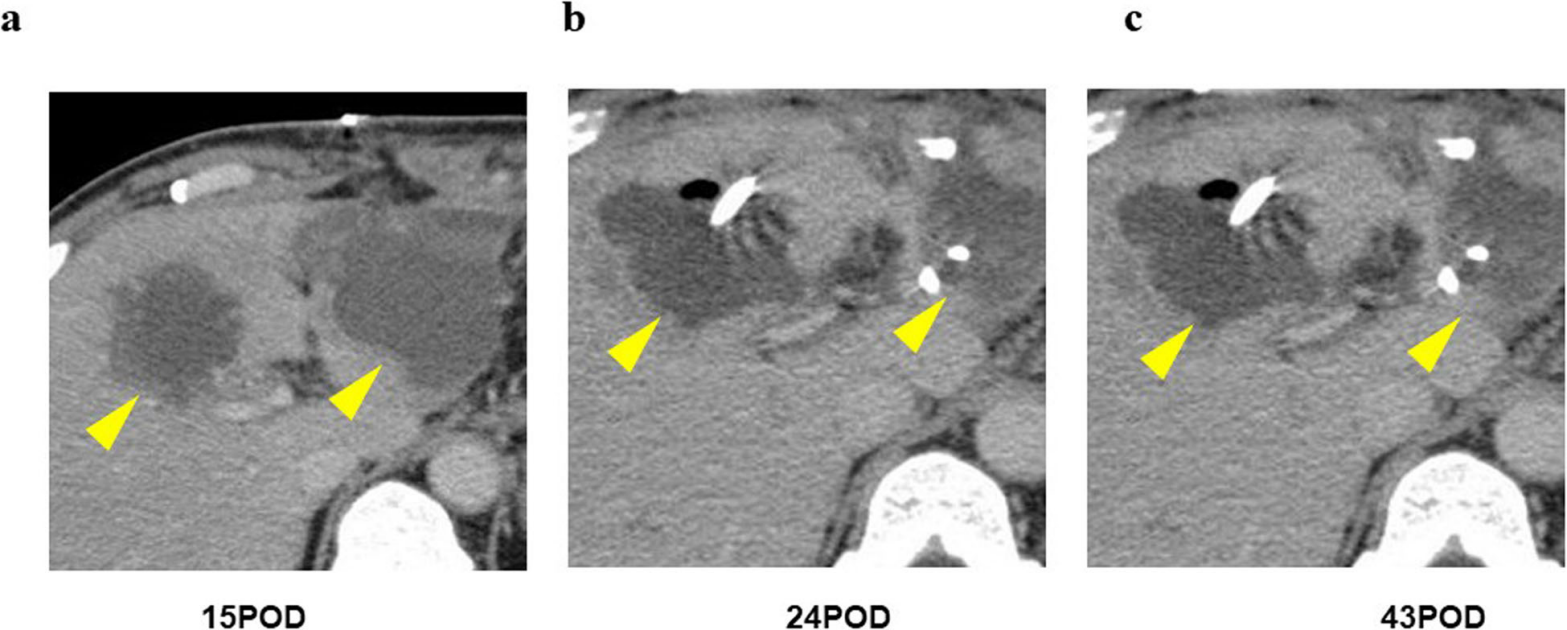
Several time drainage for multiple liver abscesses after operation

**Fig. 3:**
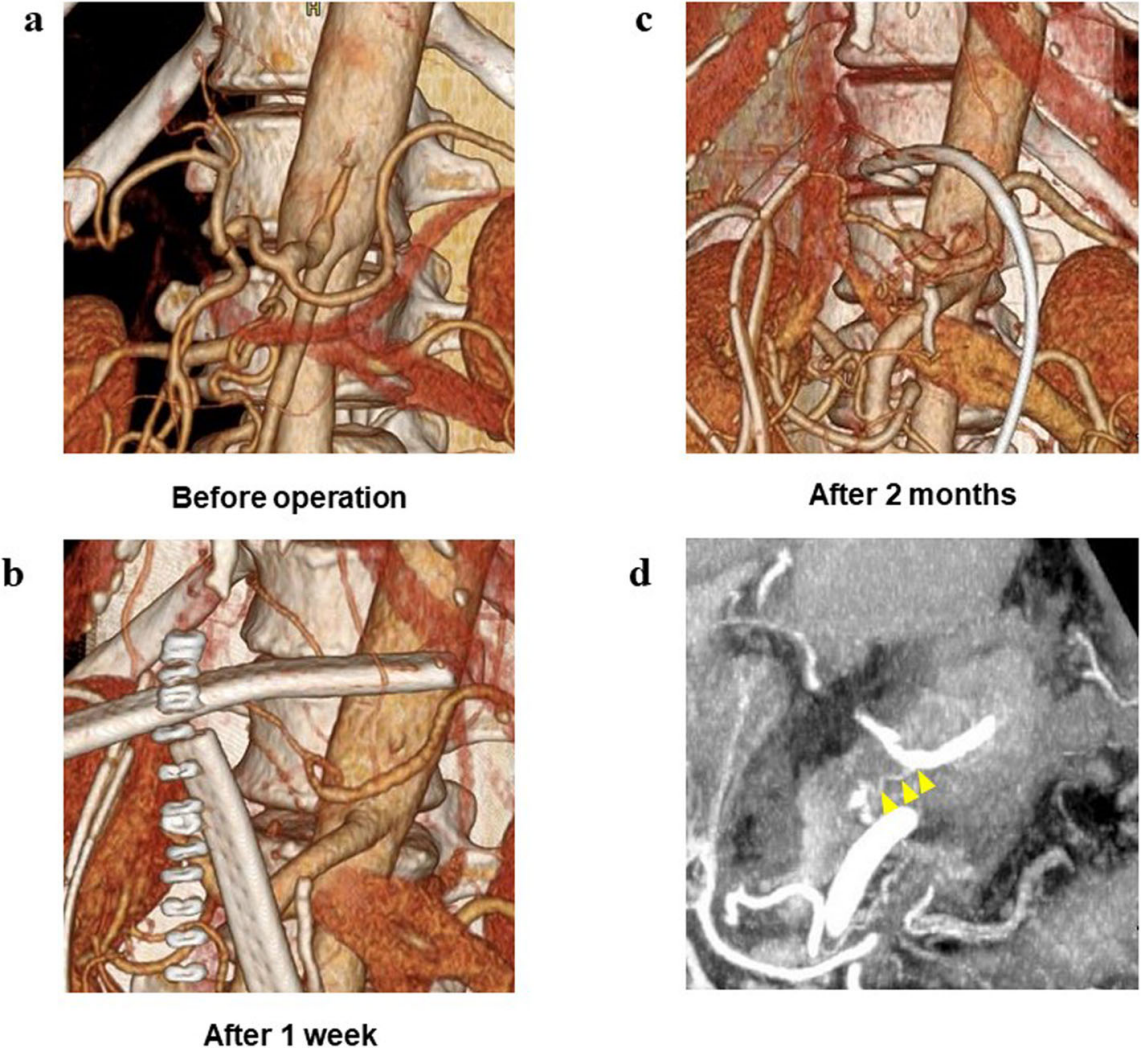
Time course of 3D CT angiography. **a** Preoperative CT; stenosis of the root of celiac artery. **b** CT at 1 week after the operation; absence of the root of celiac artery and the reduction of liver blood flow. **c** CT at 2 months after the operation; collateral circulation from dorsal pancreatic artery with recovery of liver blood flow. **d** Dorsal pancreatic artery flowed into splenic artery (1 week after the operation)

This patient presented as a healthy male in his forties. An abdominal computerized tomography (CT) for abdominal bloating and vomiting showed acute pancreatitis and stenosis of the duodenum. Conservative treatment was not successful, and he was subsequently introduced to our department, where further investigations were carried out. Blood tests showed a slight elevation of the liver deviation enzyme, but amylase and tumor markers were normal. An upper gastro-intestinal endoscopy detected redness and swelling at the descending part of the duodenum blocking the further passage of fiber. An upper gastro-intestinal examination also showed obstruction of the duodenum. An enhanced CT revealed a low-density mass surrounding the normal tissues of the pancreas and duodenum (Fig. 1a). According to magnetic resonance imaging (MRI), the mass around the pancreas and duodenum was slightly high in the intensity area in the T2-weighted image and high intensity in the diffusion-weighted area. Thus, malignancy could not be contradicted, and a pylorus-preserving pancreaticoduodenectomy (PPPD) was performed. During the operation, in the middle of reconstruction, the hepatic artery was found to be pulseless. The enhanced CT was carefully reviewed, and stenosis of the celiac trunk due to compression by the median arcuate ligament was confirmed (Fig. 1b). A three-dimensional (3D) reconstruction image of blood vessels also showed a dilated and tortuous inferior pancreaticoduodenal artery (Fig. 1c). An intra-operative diagnosis of CACS due to MALS was made. Dividing the connective tissue around the celiac trunk and elevating the patient’s blood pressure resulted in the recovery of the hepatic artery pulse. After the operation, vasopressor therapy (target: sBP 140–160 mmHg) was performed to preserve the blood flow. Prostaglandine E1 (PGE1) was also used as it contributes to dilation of the hepatic artery as well as increasing collateral circulation. In spite of the interventions, multiple liver abscesses occurred and drainage was necessary on several occasions. Drainage and antibiotic therapy were effective in causing the gradual reduction of the abscesses (Fig. 2a, b, c). An enhanced CT undertaken before the operation confirmed the presence of stenosis of the celiac trunk (Fig. 3a). One week after the operation, the roots of the celiac trunk and the proper hepatic artery were not visualized (Fig. 3b). Two months after the operation, following continuing vasopressor therapy and administration of PGE1, blood flow of the hepatic artery was recovered by growing collateral blood flow (Fig. 3c). Splenic arterial flow was good at both pre- and postoperative CT angiography. Furthermore, dorsal pancreatic artery, which was branched from SMA, flowed into the splenic artery (Fig. 3d). Because this collateral flow from dorsal pancreatic artery played important roles, ischemic complications of the stomach did not occur in this case. The patient was discharged from the hospital at 295 post-operative days.

## Case presentation 2

**Fig. 4:**
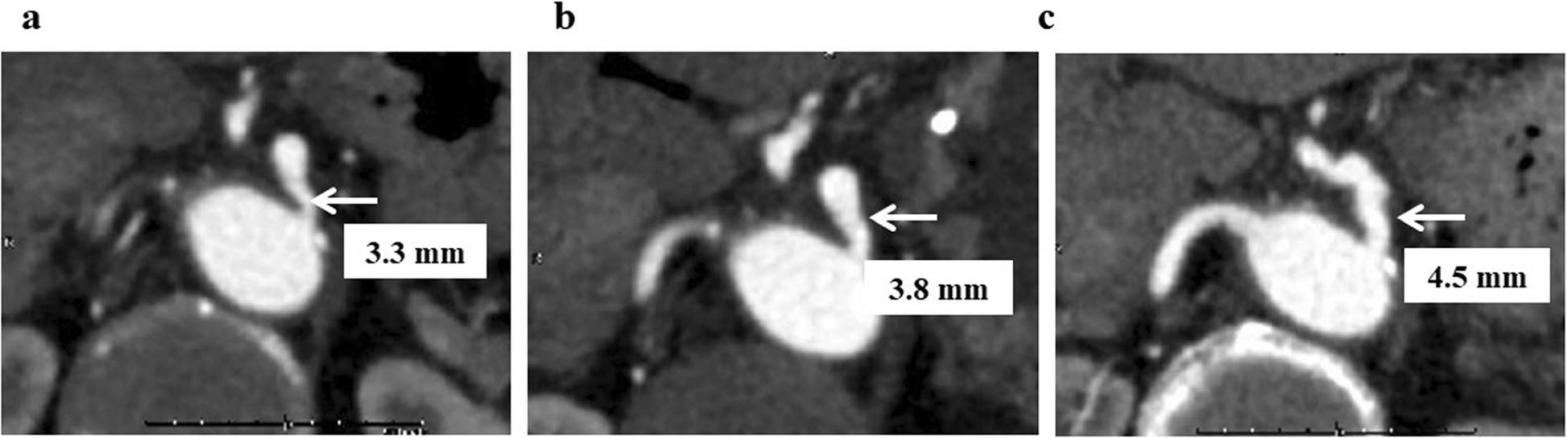
CACS due to sclerosis (case2). Improvement of celiac artery stenosis by balloon angioplasty **a** 3.3 mm, **b** 3.8 mm, and **c** 4.5 mm

This patient was a female in her seventies who had visited a nearby doctor complaining of anorexia. A blood test showed jaundice, and a CT revealed a pancreas head tumor. An ENBD tube was inserted, and then the result of pancreatic duct brushing cytology was class V. The patient was introduced to our department for an operation on the pancreas head cancer. A pre-operative CT angiography showed stenosis of the celiac trunk (Fig. 4a), and there was a calcification at the root of the celiac trunk. This was diagnosed as CACS because of the arteriosclerosis. Balloon angioplasties were performed twice before the operation, and the improvement of the stenosis was confirmed (Fig. 4b, c). While waiting for the operation, the patient underwent neo-adjuvant chemotherapy using gemcitabine and paclitaxel for four courses. It took 3 months from the initial visit to the operation, but there was no elevation of tumor markers or enlargement of the tumor size.

A sub-total stomach preserving PD (SSPPD) was performed, and there were no post-operative complications. The patient was discharged from the hospital at 21 post-operative days.

## Case presentation 3

**Fig. 5:**
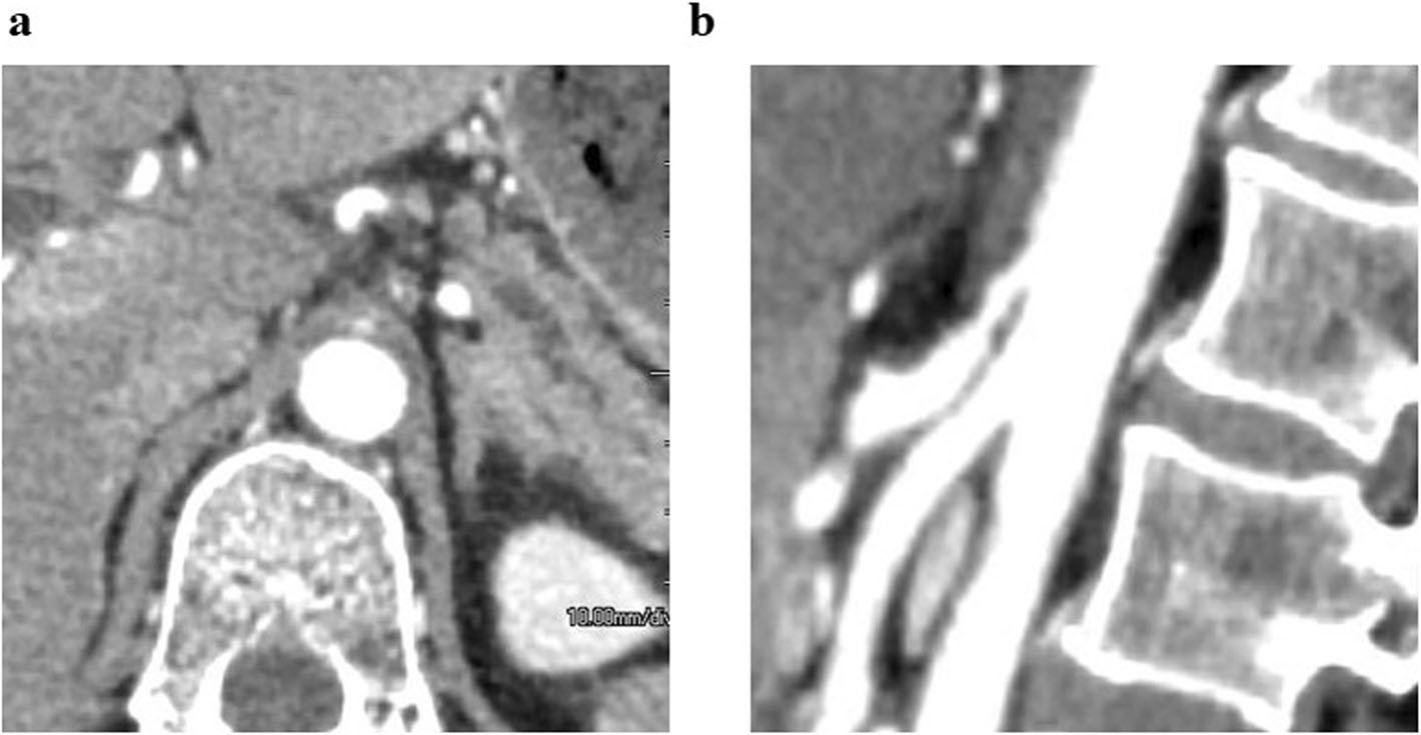
CACS due to MALS (case 3). Preoperative enhanced CT

**Fig. 6:**
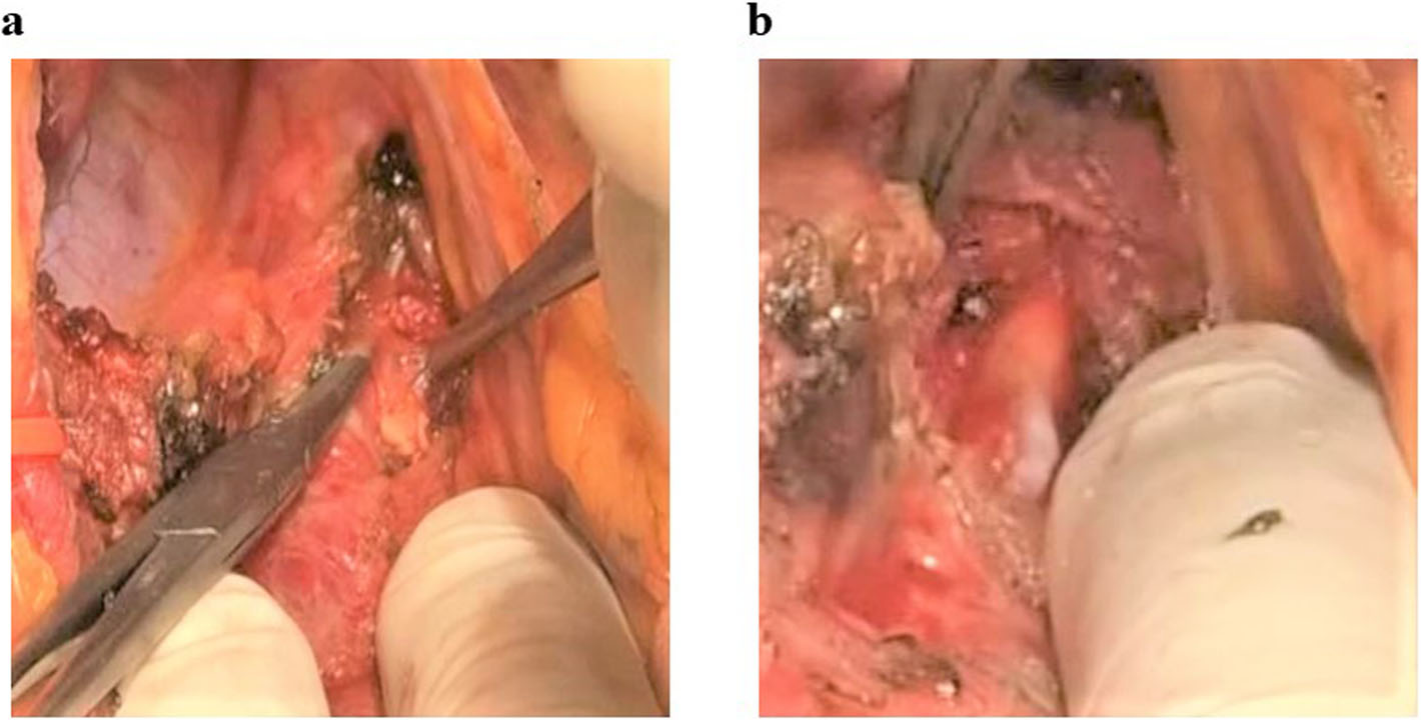
Operative findings (case 3). MAL division of the root of celiac artery

**Fig. 7:**
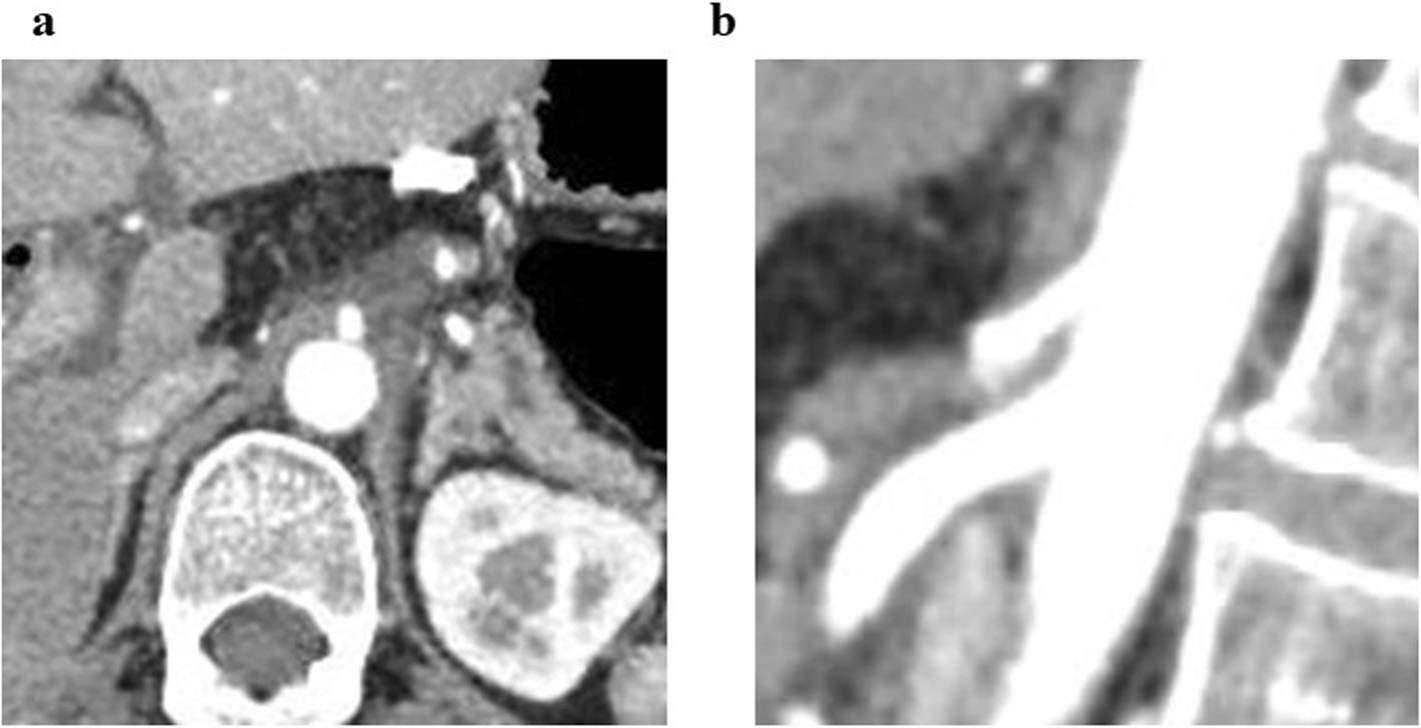
CT at postoperative day7 (case 3). Disappearance of celiac artery stenosis

The patient was a female in her fifties who was introduced to our department because of jaundice and stenosis of the inferior bile duct. Pancreatic juice cytology was class IV, so an operation was scheduled for pancreatic head cancer. A pre-operative CT angiography showed stenosis of the celiac trunk because of the compression by the median arcuate ligament and the tortuous nature of the inferior pancreaticoduodenal artery (Fig. 5a, b). MALS was diagnosed pre-operatively, and a SSPPD was performed. During the operation, the median arcuate ligament was released and improvement of the hepatic blood flow confirmed (Fig. 6a, b). Stenosis of the celiac trunk was improved as shown in the post-operative CT angiography (Fig. 7a, b). There were no post-operative complications, and the patient was discharged from the hospital at 15 post-operative days.

## Discussion

Three cases of PD co-morbid with CACS treated at our department were the subject of this study. One case, which could not be diagnosed with CACS before being operated on, had a hard post-operative course because of complication caused by ischemia of the celiac region. Conversely, the two cases, which could be diagnosed as CACS before the operation, had a good post-operative course because of pre-operative or intra-operative intervention.

When the patient is to undergo a planned PD, pre-operative diagnosis of CACS is very important. A CT angiography, which has a sensitivity of 96% and a specificity of 92%, is useful for diagnosing CACS [1]. Specific findings of CACS were already reported: (1) hooked appearance, (2) minimal post-stenotic dilatation as shown in Figs. 1b and 5b, and (3) focal calcified plaque [6]. In both MALS and sclerosis, the clues for diagnosis of CACS at CT angiography are (1) prominent collateral vessels in the pancreatic head, which are originated from SMA, and (2) dilatation of GDA or anterior superior/inferior pancreaticoduodenal artery (ASPDA/AIPDA). Since these characteristic findings of CACS may not be identified on axial images alone, 3D imaging also allows identification of CACS. Therefore, the surgeons should carefully check whether there are such collateral vessels or dilated vessels form SMA or not.

**Fig. 8:**
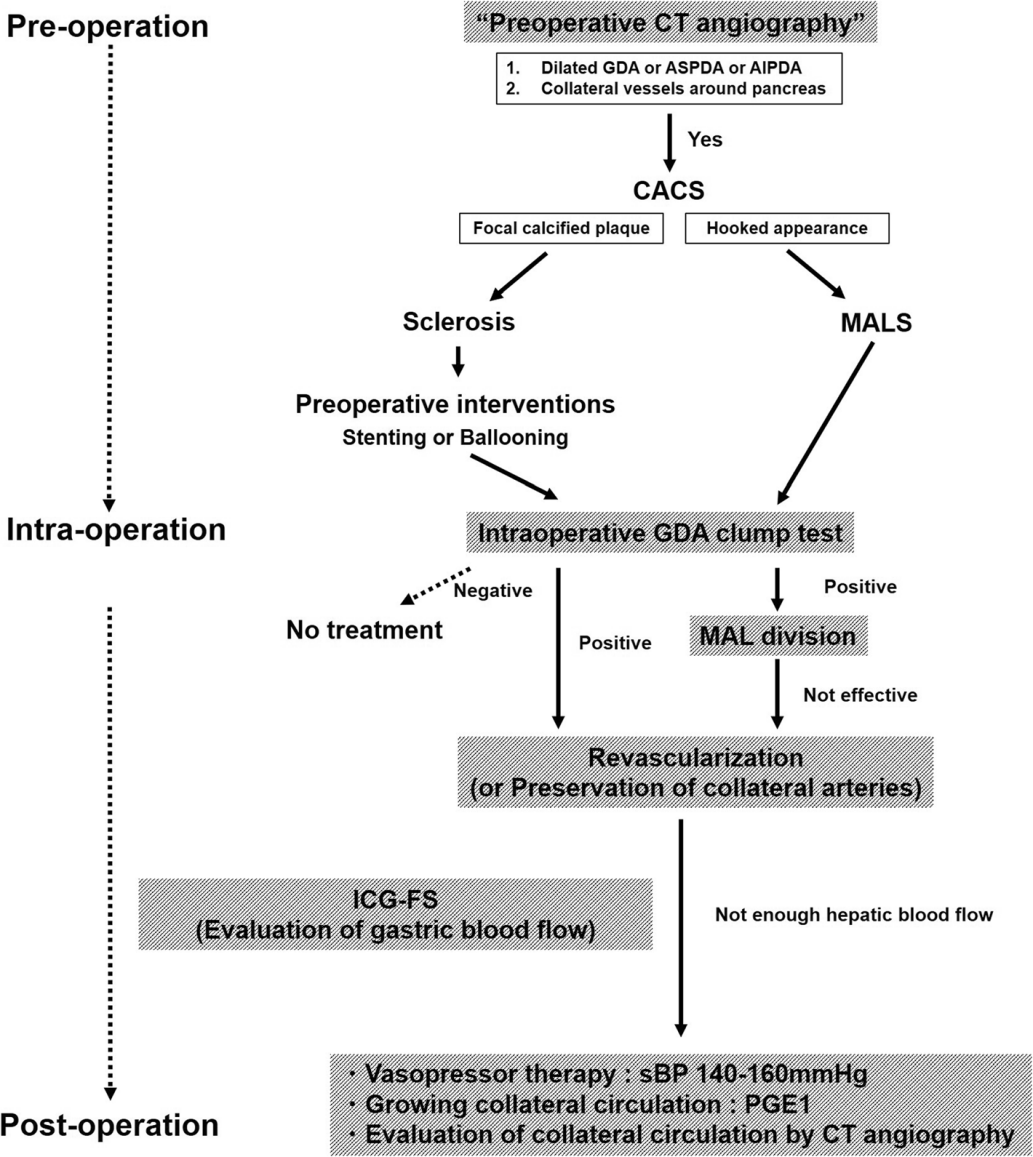
The algorithm for diagnose and treatment of CACS

The intervention for CACS is different for each case depending on the cause. Table 1 [1, 3, 7, 8–16] and Table 2 [1, 3–5, 7, 17–27] show previous reports of PD co-morbid with CACS. The cases featured in Table 1 have CACS because of sclerosis. If the reason for the diagnosis of CACS is sclerosis, there are four treatment strategies: (1) preservation of collateral arteries or GDAs, (2) arterial revascularization, (3) angioplasty by ballooning, and (4) angioplasty by stenting. Conversely, if the reason for CACS is MALS (Table 2), a GDA clamp test should be performed. When the GDA clamp test is negative, no treatment can be considered. At first, when the GDA clamp test is positive, releasing of the median arcuate ligament should be performed. And then, if improvement of the liver blood flow cannot be confirmed, re-vascularization should be considered. Even in the case with very weak palpation of hepatic artery like our case 1, we should not hesitate to release the median arcuate ligament. It was reported Doppler ultrasonography during GDA clump test is useful [1, 7, 17]; however, there were no objective criteria for releasing the median arcuate ligament or for additional hepatic arterial reconstruction. In our case 1, ischemic complications of the stomach did not occur fortunately. The indocyanine green fluorescence imaging system (ICG-FS) was reported as a useful devise which can evaluate real-time blood flow of various organs [28]. Intraoperative use of ICG-FS might be recommended for the evaluation of gastric arterial flow. It is also important to evaluate postoperative collateral circulation by CT angiography. As summarized above, Fig. 8 shows the algorithm for diagnosis and treatment of CACS.

**Table 1:** Summary of CACS caused by sclerosis on previous literature

Year	Author	Number	Procedure	Complication
1981	Thompson	*n* = 2	Splenic—SMA anastomosis	Uneventful
1988	Miyata	*n* = 1	Infrarenal Ao—CHA bypass	Uneventful
1988	Noguchi	*n* = 1	PTA	Uneventful
1993	Chikamori	*n* = 1	GDA—PIPD anastomosis	Uneventful
1995	Ii	*n* = 1	Ao—CHA bypass	Uneventful
1998	Berney	*n* = 11	2: GDA preservation 2: Ao—CHA bypass 1: CA reimplantation	1: Pancreatico-jejunal anastomotic leak
			5: No treatment	2: Liver ischemia 1: Pancreatico-jejunal anastomotic leak
2005	Kanazaka	*n* = 1	Preservation of collateral arteries	Pseudaneurysm→late hemorrhage
2005	Hayashibe	*n* = 1	Ao—CHA bypass	Uneventful
2005	Nara	*n* = 2	1:MCA—RGEA anastomosis 1: Preservation of replaced RHA	Uneventful
2006	Halazun	*n* = 1	CA stenting	Uneventful
2009	Gaujoux	*n* = 2	1: CA stenting 1: Ao—CHA bypass	Uneventful
2016	Sasaki	*n* = 1	Right common iliac artery—CHA bypass	Uneventful

**Table 2:** Summary of CACS caused by MALS on previous literature

Year	Author	Number	Procedure	Complication
1981	Fortner	*n* = 2	MAL division	Uneventful
1990	Kohler	*n* = 1	MAL division	Uneventful
1998	Okamura	*n* = 1	No treatment	Uneventful
1998	Berney	*n* = 2	MAL division	Uneventful
2003	Kawaguchi	*n* = 1	CA saphenous patch	Uneventful
2003	Hasegawa	*n* = 1	CA stenting	Uneventful
2004	Kurosaki	*n*-3	MAL division	Uneventful
2005	Shima	*n* = 1	JA—GDA anastomosis	Uneventful
2005	Nara	*n* = 1	MAL division	Uneventful
2007	Farma	*n* = 14	11: MAL division 1: Ao—CA bypass 1: Ao—CHA bypass	1: Liver abscess and biliary anastomotic leak Uneventful Uneventful
2007	Nakano	*n* = 1	MAL division	Uneventful
2009	Gaujoux	*n* = 55	32: No treatment 23: MAL division	32: Uneventful 1: CA thrombosis 1: Stomach ischemia
2011	Saito	*n* = 1	MAL division	Uneventful
2012	Sugae	*n* = 12	1: No treatment 8: MAL division 2: IPDA—GDA anastomosis 1: Preservation of collateral arteries	1: Ischemic of pancreas tail, spleen, and residual stomach Uneventful Uneventful Uneventful
2016	Park et al.	*n* = 2	1: CHA—Ao bypass 1: CA stenting	Uneventful
2018	Yamamoto	*n* = 1	MAL division	Uneventful

As limitation of this report, there were already many case reports of CACS, and its treatment is shown in Tables 1 and 2. In this report, we underwent a case with severe post-operative complications without enough knowledge of CACS. Based on this critical clinical experience, following two cases had good post-operative courses without any postoperative complications by appropriate pre-operative intervention or intra-operative MAL division. In this article, we have summarized three CACS cases in our own facility over the past decade.

## Conclusion

In conclusion, post-operative complications due to CACS are preventable by pre-operative diagnosis and appropriate interventions.

## Data Availability

The datasets supporting the conclusions of this article are included within the article and its additional files.

## Abbreviations

CACS: Celiac axis compression syndrome
MALS: Median arcuate ligament syndrome
GDA: Gastroduodenal artery
SMA: Superior mesenteric artery
PD: Pancreatoduodenectomy
PPPD: Pylorus-preserving PD
SSPPD: Sub-total stomach preserving PD
PGE1: Prostaglandine E1
GDE: Delayed gastric empty
MAL: Median arcuate ligament
ASPDA: Anterior superior pancreaticoduodenal artery
AIPDA: Anterior inferior pancreaticoduodenal artery
ICG-FS: ICG fluorescence imaging system
